# Fecal Microbiota Transplantation in Alzheimer’s Disease: Mechanistic Insights Through the Microbiota–Gut–Brain Axis and Therapeutic Prospects

**DOI:** 10.3390/microorganisms13081956

**Published:** 2025-08-21

**Authors:** Jiayu Ren, Qinwen Wang, Hang Hong, Chunlan Tang

**Affiliations:** School of Public Health, Health Science Center, Ningbo University, Ningbo 315211, China

**Keywords:** fecal microbiota transplantation, Alzheimer’s disease, microbiota–gut–brain axis

## Abstract

Alzheimer’s disease (AD), a prevalent neurodegenerative disorder in the aging population, remains without definitive therapeutic solutions. Emerging insights into the gut microbiota (GM) and its bidirectional communication with the central nervous system(CNS) through the microbiota–gut–brain axis (MGBA) have unveiled potential correlative mechanisms that may contribute to AD pathogenesis, though causal evidence remains limited. Dysregulation of GM composition (dysbiosis) exacerbates AD progression via neuroinflammation, amyloid-β (Aβ) deposition, and tau hyperphosphorylation (p-tau), while restoring microbial homeostasis presents a promising therapeutic strategy. Fecal microbiota transplantation (FMT), a technique to reconstitute gut ecology by transferring processed fecal matter from healthy donors, has demonstrated efficacy in ameliorating cognitive deficits and neuropathology in AD animal models. Preclinical studies reveal that FMT reduces Aβ plaques, normalizes tau phosphorylation, suppresses inflammasome activation, and restores microglial homeostasis through modulation of microbial metabolites and immune pathways. Although clinical evidence remains limited to case reports and small-scale trials showing potential therapeutic effect, safety concerns regarding long-term effects and protocol standardization necessitate further investigation. This review synthesizes current knowledge on GM–AD interactions, evaluates FMT’s mechanistic potential, and discusses challenges in translating this ancient practice into a cutting-edge AD therapy. Rigorous randomized controlled trials and personalized microbiota-based interventions are imperative to advance FMT from bench to bedside.

## 1. Introduction

Alzheimer’s disease (AD), the most prevalent neurodegenerative disorder worldwide, currently affects over 35 million individuals and is projected to triple by 2050 due to global population aging [1]. Characterized by progressive cognitive decline and neuropathological hallmarks including amyloid-β (Aβ) plaques, neurofibrillary tangles (NFTs) of hyperphosphorylated tau, and chronic neuroinflammation, AD remains incurable with existing therapies offering only symptomatic relief [2]. Current pharmacological interventions for AD primarily comprise symptomatic treatments, such as cholinesterase inhibitors (e.g., donepezil) and memantine, which provide modest cognitive improvement but fail to halt disease progression [3,4]. Emerging disease-modifying therapies (DMTs)—notably anti-amyloid β monoclonal antibodies like lecanemab—have demonstrated efficacy in slowing cognitive decline during early AD stages; however, their long-term benefits and safety profiles remain controversial [5]. The failure of more than 200 clinical trials targeting Aβ and tau pathology in the past decade [6] has necessitated a paradigm shift toward understanding modifiable risk factors, particularly the emerging role of gut–brain axis dysregulation in AD pathogenesis.

Recent breakthroughs in microbiome research have unveiled the gut microbiota (GM) as a critical regulator of neurological health through the microbiota–gut–brain axis (MGBA) [7]. Mounting evidence indicates that GM dysbiosis precedes AD onset and correlates with disease severity [8], mediated through multiple interconnected mechanisms: (1) microbial metabolite-mediated neuroinflammation [9], (2) dysregulation of neurotransmitter systems (serotonin, GABA) [10], (3) endocrine disruption via hypothalamus–pituitary–adrenal (HPA) axis overactivation [11], and (4) compromised intestinal and blood–brain barrier (BBB) integrity [12]. Notably, the “gut-first” pathological model posits that age-related GM changes may initiate peripheral inflammation that subsequently breaches theBBB, creating a self-perpetuating cycle of neurodegeneration [13].

In this context, fecal microbiota transplantation(FMT) has emerged as a revolutionary therapeutic strategy. While traditionally used for *Clostridioides difficile* infection, preclinical studies demonstrate FMT’s capacity to remodel gut ecosystems, reduce Aβ burden by 40–60% in AD mouse models [14], and reverse tau hyperphosphorylation (p-tau) [15]. The GUT-PARFECT trial’s recent success in Parkinson’s disease [16] and case reports of cognitive improvement in AD patients [17] further support its translational potential. However, critical knowledge gaps persist regarding donor selection criteria, long-term safety profiles, and the precise mechanisms underlying FMT’s neuromodulatory effects.

This review offers the first comprehensive analysis of FMT dual regulatory role in AD progression. Additionally, we propose a novel “multi-hit” model that integrates neural, endocrine, metabolic, and immunological pathways. This model elucidates how GM modulation influences AD pathology and aims to bridge the translational gap between animal studies and clinical applications.

## 2. Gut Microbiota and Microbial–Gut–Brain Axis Mechanisms

### 2.1. Gut Microbiota

The human body hosts a vast array of microorganisms, collectively termed the microbiota, which reside in various niches including the urogenital tract, respiratory system, skin, oral cavity, and most abundantly, the GIT. It is estimated that 95% of these symbiotic microorganisms are found in the gut [18], comprising a diverse ecosystem known as the GM, which contains approximately 100 trillion microorganisms, including bacteria, fungi, phages, and viruses [19]. In a healthy state, the gut is dominated by the phyla *Firmicutes* and *Bacteroidetes*, which together account for more than 90% of the microbial population. Based on the interaction of these bacteria with the host, the GM can be categorized into three groups: symbiotic beneficial bacteria such as *Bifidobacteria* and *Lactobacilli*; commensal opportunistic pathogens like *Escherichia* and *Enterococcus*; and pathogenic bacteria such as *Proteus* and *Salmonella typhi* [20].

The GM is integral to human health, participating in metabolic processes, immune system constitution, nutrient absorption, detoxification, vitamin synthesis, and pathogen exclusion [21]. These microorganisms coexist in a delicate balance, with their interdependence maintaining homeostasis in healthy individuals. However, this balance shifts with age. Mitsuoka [22] observed that as age increases, the gut Bifidobacteria decrease, while Clostridium, Streptococcus, and Enterobacteriaceae become more abundant. Elderly individuals exhibit a reduced abundance of probiotics and an increase in proinflammatory opportunistic pathogens compared to younger adults [23]. A diminished diversity and altered abundance of GM lead to dysbiosis, a state that can precipitate metabolic disorders and is closely associated with the development of gastrointestinal, immune system, metabolic, and nervous system diseases [24]. Dysbiosis may serve as a disease indicator, and its intervention could be a crucial strategy for disease prevention and treatment.

Research indicates that approximately 45% of bacterial species detected in the gut originate from the oral cavity, underscoring the significant contribution of oral microbiota to intestinal microbial diversity [25]. Oral dysbiosis refers to the disruption of microbial community equilibrium in the oral niche, characterized by overgrowth of pathobionts (e.g., *Porphyromonas gingivalis*, *Fusobacterium nucleatum*) and depletion of beneficial taxa (e.g., *Streptococcus salivarius*, *Lactobacillus* spp.) [26]. Salivary microbiome analyses reveal that *Porphyromonas gingivalis*—a recognized periodontal pathogen—can survive gastric acidity and proliferate upon intestinal translocation, thereby driving gut dysbiosis [27]. Lipopolysaccharide (LPS) produced by *P. gingivalis* enters systemic circulation, triggering low-grade systemic inflammation, insulin resistance, and increased intestinal permeability [28]. Moreover, *Fusobacterium nucleatum*, another periodontitis-associated pathobiont, translocates to the gut, disrupting microbial composition and impairing immune and metabolic regulation [29]. Collectively, oral dysbiosis is not an isolated event; through dynamic crosstalk with the gut microbiome, it synergistically accelerates cognitive decline via immunologic, metabolic, and neural pathways.

### 2.2. MGBA Mechanisms of AD

#### 2.2.1. Neural Pathways

**Figure 1:**
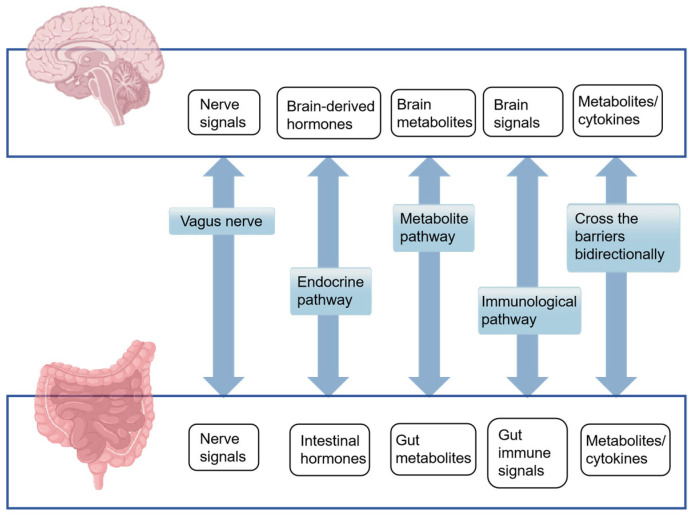
Five major pathways of the human microbiota–gut–brain axis mechanism.

The MGBA is a complex and dynamic communication network that encompasses the bidirectional interactions between the GM and the central nervous system (CNS). This intricate axis facilitates the influence of the GM on CNS activity through a multitude of pathways, which include neural, endocrine, metabolic, and immune signaling, as well as the modulation of the gut mucosal barrier and the BBB (Figure 1). These interactions play a pivotal role in regulating brain function, impacting the nervous system, and shaping cognitive abilities.

The human body is equipped with a vast network of enteric neurons and the vagus nerve, which are integral to the communication between the gut and the brain. The vagus nerve, a key component of the parasympathetic nervous system, is composed of approximately 80% afferent fibers and 20% efferent fibers [30]. This nerve serves as a conduit connecting the GIT to the brain and is a vital element of the MGBA. Information originating from the gut is conveyed to the brain via enteric neurons and the vagus nerve, enabling the brain to regulate and modulate gut functions.

**Figure 2:**
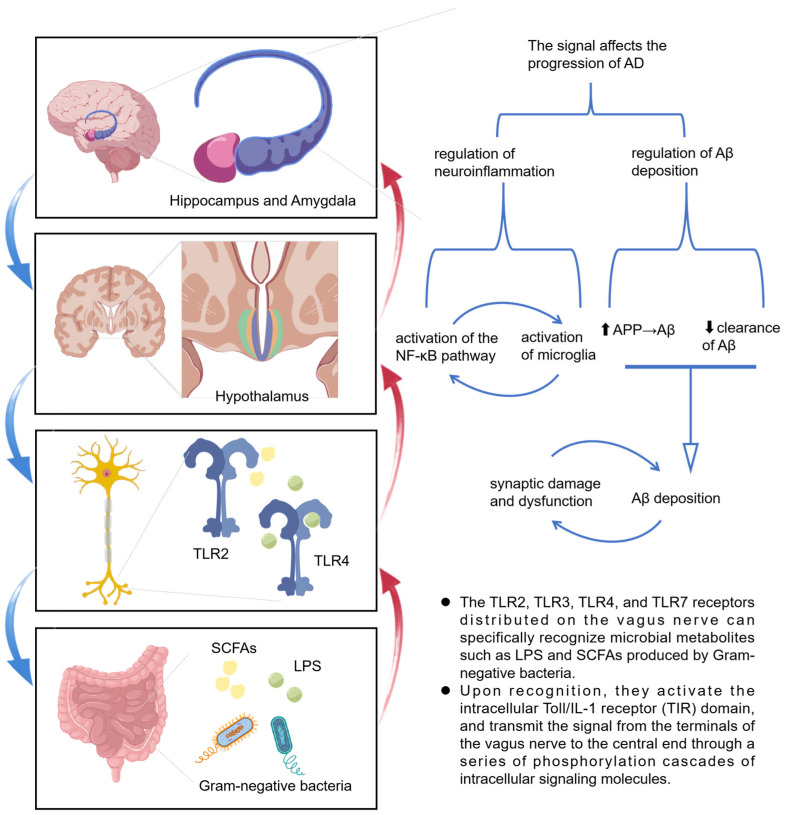
Schematic diagram of vagus nerve signaling stimulated by microbial metabolites (SCFAs, LPS) in Alzheimer’s disease progression. The upward arrow “⬆” indicates that the expression of amyloid precursor protein (APP) is increased, which promotes the formation of amyloid-β (Aβ), while the downward arrow “⬇” indicates that the clearance of Aβ is decreased; these two mechanisms together lead to the accumulation of Aβ.

Research by Liu et al. [31] demonstrated that the administration of *Lacticaseibacillus rhamnosus* to mice led to a reduction in anxiety behaviors, a decrease in HPA axis activity, and an increase in exploratory behavior. However, these effects were not observed in mice with a severed vagus nerve, thereby underscoring the essential role of the vagus nerve in mediating the communication between gut bacteria and the brain. The GM can influence the vagus nerve through two primary mechanisms. Firstly, it can directly interact with the enteric nervous system, thereby activating the associated vagus nerve. The local signals generated by this interaction are transmitted through sensory neural circuits to brain regions responsible for cognition, emotion, fear, and anxiety [32], thereby modulating brain function and potentially impacting the progression of AD. Secondly, the GM produces metabolites such as short-chain fatty acids (SCFAs) and lipopolysaccharides(LPS) that can stimulate the vagus nerve. The vagus nerve’s surface is equipped with toll-like receptors (TLRs) 2, 3, 4, and 7, which are capable of detecting signals from the GM [33,34], establishing a bidirectional communication pathway between the gut and the brain (Figure 2).

#### 2.2.2. Endocrine Pathway

The endocrine pathway is a critical component of the MGBA, through which the GM can exert significant influence on neurotransmitter balance and brain function, potentially impacting the development and progression of AD. Neurotransmitters, the chemical messengers of the nervous system, are categorized as either excitatory or inhibitory. An imbalance between these two types can lead to abnormal neural activity, which may contribute to the pathology of AD [35]. The GM plays a dual role in this context: it can directly produce precursors to neurotransmitters and can also modulate their synthesis through dietary metabolism, thereby influencing neurotransmitter levels via neuroendocrine pathways. Notable neurotransmitters in this context include 5-hydroxytryptamine (5-HT, also known as serotonin) and γ-aminobutyric acid (GABA). More than 90% of the body synthesis of 5-HT, a neurotransmitter closely associated with mood regulation, occurs in enterochromaffin cells. The GM can interact with colonic epithelial cells to regulate 5-HT levels in the colon and serum. Furthermore, it can modulate the gene expression of tryptophan hydroxylase 1, the rate-limiting enzyme in 5-HT synthesis, thereby influencing 5-HT levels and brain function [10]. 5-HT has been shown to have a positive effect on cognitive function in AD patients. Direct injection of 5-HT into the hippocampus of mice has been observed to reduce levels of Aβ in the cerebrospinal fluid [36]. The GM also plays a role in GABA synthesis. Microbiota such as *Bifidobacterium* and *Lactobacillus* can convert glutamate to GABA. With aging, the abundance of these beneficial bacteria tends to decrease, leading to reduced GABA production. This can disrupt the balance between the sympathetic and parasympathetic nervous systems, potentially leading to overactivity of the brain. Excessive excitation can damage neurons, or even lead to the loss of neurons, ultimately affecting memory formation and maintenance [37]. Additionally, the GM can modulate brain and gut function through the HPA axis [11]. The HPA axis is a crucial part of the neuroendocrine system that responds to stress. Under stress, the HPA axis can become overactivated, leading to the overproduction of corticosteroids and alterations in GM composition. These changes can influence the CNS by modulating stress hormones, resulting in elevated corticosteroid levels that can affect the structure of the hippocampus and amygdala, thereby impairing cognitive function [11,38]. In summary, the endocrine pathway within the MGBA provides a mechanism through which the GM can directly and indirectly influence neurotransmitter synthesis and brain function.

#### 2.2.3. Microbial Metabolite

Microbial metabolites and bioactive peptides are key players in the MGBA, exerting significant influence on the progression of AD. Among these metabolites, LPS and SCFAs produced by the GM have been implicated in shaping the disease’s trajectory.

Ageing is associated with an increase in Gram-negative bacteria, particularly *Bacteroides* in the GM, leading to heightened endotoxin LPS production. LPS is known to trigger neuroinflammatory responses by engaging TLR4, which in turn leads to the overactivation of microglia and amplifies their cytotoxic effects. This can result in irreversible pathological changes characteristic of AD and cognitive memory impairments [39]. Furthermore, LPS can induce the transformation of astrocytes into neurotoxic astrocytes, contributing to neuronal and oligodendrocyte death [40]. Clinical studies have revealed higher LPS levels in the brains of AD patients compared to age-matched controls, underscoring the link between excessive LPS and AD pathogenesis [41].

Short-chain fatty acids (SCFAs, e.g., butyrate, propionate)—produced by GM fermenting dietary fiber—exert neuroprotection via epigenetic regulation, mitochondrial homeostasis maintenance, and immunomodulation. In 5xFAD transgenic AD mice, butyrate inhibits histone deacetylase (HDAC), upregulates brain-derived neurotrophic factor (BDNF) expression, and enhances synaptophysin (SYP) activity, improving spatial memory (reduction in Morris water maze escape latency) [42]. Propionic acid activates GPR41/GPR43 receptors to regulate neuronal mitochondrial dynamics by ① downregulating Drp1 to reduce excessive fission, and ② activating the PINK1/PARKIN pathway to clear damaged mitochondria via mitophagy, decreasing Aβ-induced neuronal apoptosis [43]. Notably, butyrate-producing bacteria (e.g., *Faecalibacterium prausnifera*) are depleted in AD patients and models, while exogenous butyrate restores blood–brain barrier integrity and suppresses microglial IL-6 secretion [44,45]. Research has indicated significantly reduced SCFAs levels in the intestines of AD patients [8]. A deficiency in SCFAs may, therefore, lead to impaired Aβ clearance, contributing to the pathogenesis of AD [46,47].

Secondary bile acids (e.g., deoxycholic acid, DCA) suppress neuroinflammation by activating the farnesoid X receptor (FXR). However, reduced DCA/CA (cholic acid) ratios in AD patients impair FXR signaling, resulting in an increase in microglial IL-1β secretion [48]. Trimethylamine N-oxide (TMAO)—derived from gut microbial metabolism of dietary choline—disrupts blood–brain barrier tight junctions, promotes tau hyperphosphorylation, and activates the NLRP3 inflammasome, correlating with elevated mild cognitive impairment (MCI)-to-AD conversion risk [48,49]. Furthermore, polyunsaturated fatty acid metabolites like prostaglandin E2 (PGE2) are enriched in the AD gut, exacerbating neuroinflammation and Aβ generation via the C/EBPβ/AEP pathway. Aspirin-mediated inhibition of PGE2 synthesis ameliorates cognitive deficits in 5xFAD mice [45,50].

Tryptophan metabolites exert bidirectional effects on AD pathogenesis through kynurenine and indole derivative pathways. In the kynurenine pathway, upregulated tryptophan 2,3-dioxygenase (TDO) in the AD gut leads to accumulation of neurotoxic quinolinic acid, which activates NMDA receptors and induces oxidative stress (hippocampal lipid peroxidation ↑ 48%). Quinolinic acid crosses the blood–brain barrier, triggering microglial TNF-α release and exacerbating Aβ deposition [48]. Conversely, in the indole pathway, probiotics (e.g., *Lactobacillus fermentum*) convert tryptophan to indole-3-propionic acid (IPA). IPA activates the aryl hydrocarbon receptor (AhR) to inhibit NF-κB signaling, reducing Aβ plaques and phosphorylated tau in *APP/PS1* mice. It also preserves GABAergic synaptic function, improving cognitive impairment [42,49]. Interventions combining Qingke β-glucan with *lactobacilli* significantly lower the kynurenine/tryptophan ratio, reversing neurotransmitter imbalances in AD models [42].

In summary, microbial metabolites such as LPS and SCFAs are integral to the MGBA and can significantly influence AD progression.

#### 2.2.4. Immunological Pathway

**Figure 3:**
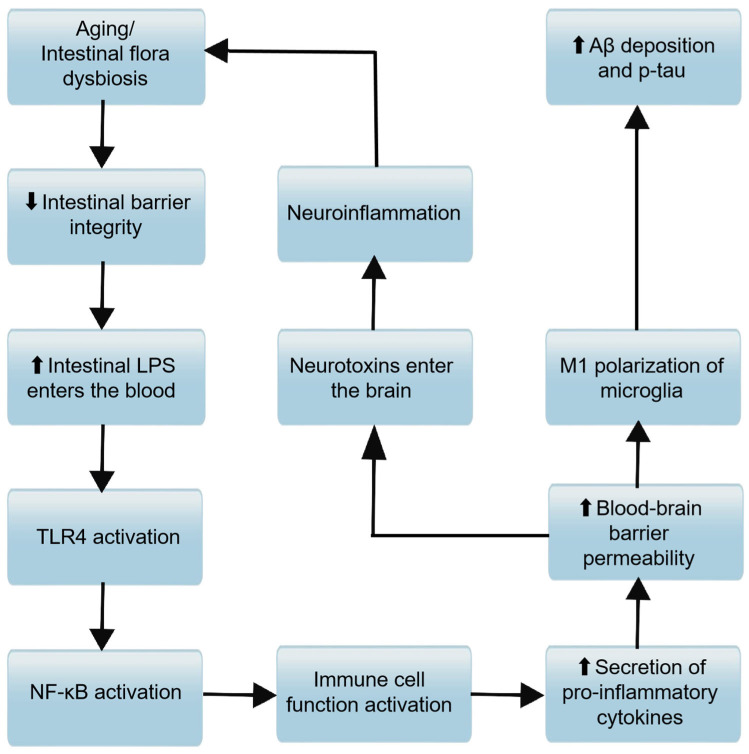
Molecular mechanism associations between immune pathways and barrier functions in the progression of AD. Aging or intestinal flora dysbiosis downregulates intestinal barrier integrity (the downward arrow “⬇” denotes decreased integrity). Reduced intestinal barrier integrity allows more intestinal lipopolysaccharide (LPS) to enter the bloodstream (the upward arrow “⬆” denotes increased LPS entry). NF-κB activation promotes immune cell function activation, leading to increased secretion of pro-inflammatory cytokines.Elevated pro-inflammatory cytokines enhance blood-brain barrier permeability (the upward arrow “⬆” denotes increased permeability). Increased blood-brain barrier permeability facilitates M1 polarization of microglia; M1-polarized microglia then contribute to enhanced amyloid-β (Aβ) deposition and phosphorylation of tau (p-tau) (the upward arrow “⬆” denotes increased levels). Meanwhile, heightened blood-brain barrier permeability also enables neurotoxins to enter the brain; neurotoxins induce neuroinflammation, and neuroinflammation further reinforces “aging or intestinal flora dysbiosis,” forming a vicious cycle that drives AD progression.

As shown in Figure 3, the immunological pathway is a critical component of the MGBA, through which the GM plays a pivotal role in modulating host immune homeostasis and influencing the pathogenesis of AD. As we age, the GM undergoes significant changes in both quantity and composition, often favoring the proliferation of pro-inflammatory bacteria while suppressing the growth of anti-inflammatory species. These microbial shifts can activate peripheral immune cells, leading to a surge in inflammatory cytokines such as interleukin-1 (IL-1), interleukin-6 (IL-6), and tumor necrosis factor-alpha (TNF-α). A critical mediator linking these microbial shifts to amplified inflammatory responses is the NLRP3 inflammasome, a cytosolic multiprotein complex that acts as a key sensor of cellular stress. Upon activation by microbial components, aggregated Aβ, or oxidative stress—all of which are hallmarks of AD pathogenesis—the NLRP3 inflammasome triggers the cleavage of pro-caspase-1 into its active form, thereby promoting maturation and secretion of pro-inflammatory cytokines like IL-1β, a central driver of neuroinflammation in AD [51,52]. Additionally, NLRP3 activation induces pyroptosis, a pro-inflammatory form of cell death that releases intracellular contents, further exacerbating local and systemic inflammation. In the context of AD, sustained NLRP3 activation in microglia and astrocytes accelerates Aβ deposition and tau hyperphosphorylation [53], creating a vicious cycle between neuroinflammation and neurodegeneration. Notably, gut microbial metabolites such as SCFAs, whose levels decline with age-related dysbiosis, normally suppress NLRP3 activation [54], highlighting a direct link between GM dysregulation and inflammasome-mediated neuroinflammatory processes in AD. This activation can initiate a cascade of localized systemic inflammation, increase gastrointestinal permeability, and affect the CNS through the bloodstream. It can compromise the integrity of the BBB, foster neuroinflammation, and ultimately impair cognitive function [13,55]. The gut microbiome also plays a role in the selection and activity of invariant natural killer T cells (iNKT), further modulating immune responses [56]. This activation stimulates immune cells to secrete pro-inflammatory cytokines and immunoglobulins (IgM/IgA), thereby exacerbating systemic inflammation [57,58]. These pro-inflammatory cytokines can, in turn, enhance the expression of the amyloid precursor protein (APP), promoting the formation of Aβ in the hippocampus [59]. Microglia, the resident immune effector cells of the CNS, can initiate neuroinflammation in response to changes in GM metabolites, further contributing to the neuroinflammatory environment associated with AD. In summary, the immunological pathway within the MGBA is a key conduit through which the GM can influence the peripheral immune system and, consequently, the CNS. By modulating the balance of peripheral inflammatory factors, the GM can either promote or ameliorate the neuroinflammatory processes that characterize AD.

#### 2.2.5. Intestinal Wall Barrier and Blood–Brain Barrier

The integrity of the intestinal wall barrier and the BBB is integral to maintaining health and preventing disease, including AD. The MGBA encompasses these barriers, highlighting the influence of GM on their function and the subsequent impact on neurological health. Alterations in the GM’s structure can disrupt the ecological balance of the gut, leading to changes in intestinal barrier permeability. Aging is associated with increased permeability of both the intestinal mucosal barrier and the BBB, a phenomenon that can have profound implications for systemic health and neurological function. The GM is crucial for maintaining the integrity of the BBB: studies in germ-free mice have shown increased BBB permeability, possibly due to reduced expression of tight junction proteins, which are essential for maintaining the barrier’s integrity [12]. When intestinal barrier permeability is increased, pathogenic bacteria and harmful microbial metabolites, such as LPS, can translocate into the systemic circulation. LPS can directly damage endothelial cells or bind to TLR4, initiating a cascade of systemic inflammation by activating the expression of NF-κB and producing inflammatory mediators such as IL-6, TNF-α, and prostaglandin E2 [9]. This activation of the host immune defense system can lead to a state of chronic low-grade inflammation. These inflammatory mediators subsequently compromise BBB integrity, initiating a cascade of events that includes neuroinflammation, cognitive deterioration, and impaired CNS regulation of gut function. This self-perpetuating cycle creates a detrimental feedback loop that potentially accelerates AD pathogenesis and progression. Increased permeability of the BBB is indeed considered a hallmark of dysfunction in AD models [60]. In summary, the intestinal wall barrier and the BBB are critical components of the MGBA (Figure 3), with the GM playing a significant role in their maintenance. Disruptions in these barriers can lead to a cycle of inflammation and neuroinflammation, contributing to the pathogenesis of AD.

## 3. FMT for the Treatment of AD

### 3.1. FMT

#### 3.1.1. The History and Development of FMT

FMT is a therapeutic procedure with a rich historical backdrop, dating back to ancient China. During the Eastern Jin Dynasty, the physician Ge Hong detailed the use of fecal suspensions to treat conditions like food poisoning and diarrhea in his seminal work Zhou Hou Bei Ji Fang [61]. In the 16th century, the renowned Chinese physician Li Shizhen further chronicled the effectiveness of FMT in treating severe vomiting, abdominal pain, diarrhea, constipation, and other digestive ailments in *Bencao Gangmu* [62].

FMT gained significant recognition in 2013 when Zhang et al. included it in clinical guidelines for the treatment of recurrent *Clostridioides difficile* infection (CDI) in the United States [63]. The expanding field of GM research has revealed increasingly promising therapeutic applications for FMT. The scope of FMT has evolved substantially beyond its initial focus on gastrointestinal disorders to encompass a broader spectrum of conditions, including neurological and metabolic diseases. Emerging experimental evidence demonstrates that FMT can effectively modulate the GM composition and regulate both neurotransmitter systems and microbial metabolites, potentially enhancing cognitive function in AD patients [13,64]. These findings provide novel therapeutic perspectives and mechanistic insights for AD intervention strategies.

#### 3.1.2. Selection of FMT Donors

Optimal donor selection for FMT requires comprehensive multidimensional evaluation to ensure safety and efficacy. Donors must undergo rigorous health screening to exclude active infections, inflammatory bowel diseases, metabolic disorders, and malignancies. Blood and stool testing should eliminate pathogens including *Clostridioides difficile*, multidrug-resistant organisms, and SARS-CoV-2. Psychological evaluation via the Symptom Checklist-90 (SCL-90) excludes mental health disorders (e.g., anxiety, depression), while behavioral screening disqualifies individuals with high-risk sexual behaviors or recent infectious disease exposure [65]. Antibiotic usage within 12 months prior to donation—particularly broad-spectrum antibiotics—significantly reduces FMT success rates and must be strictly restricted [66]. Microbial diversity and functional integrity are paramount: donors should exhibit high α-diversity (e.g., Shannon index > 5), stable core microbiota (e.g., phyla *Bacteroidetes* and *Firmicutes*), and abundant SCFAs-producing bacteria (e.g., *Faecalibacterium prausnitzii*) [65,67]. Metagenomic sequencing must assess functional gene profiles related to carbohydrate metabolism and anti-inflammatory pathways [68]. Younger donors (aged 18–30 years) demonstrate superior clinical outcomes [69].

A subset of donors demonstrating consistently higher remission rates across multiple recipients are termed “super-donors” [70]. China’s 2022 Expert Consensus on Donor Screening and Management for FMT establishes a “five-step six-dimensional” framework integrating physiological, psychological, lifestyle, and microbiological assessments to standardize donor quality [65]. International guidelines further emphasize multiplex screening for multidrug-resistant organisms to mitigate transmission risks [71].

#### 3.1.3. Fecal Microbiota Preparation

The clinical implementation of FMT encompasses a rigorous and time-sensitive protocol. The procedure initiates with the procurement of fresh fecal material from thoroughly screened donors, followed by immediate anaerobic transport to designated FMT facilities. The microbiota preparation process utilizes sophisticated automated isolation systems (e.g., GenFMTer), employing sequential microfiltration and centrifugation techniques to achieve optimal microbial enrichment and purification, culminating in a standardized fecal suspension. This preparation is administered promptly, although cryopreservation with glycerol at −80 °C is feasible when necessary. The entire procedural workflow is optimized to comply with the “one-hour FMT protocol” [72].

#### 3.1.4. Antibiotic Pretreatment

Recipient preparation typically involves a standardized protocol of antibiotic preconditioning and bowel preparation to establish an optimal environment for microbial engraftment [73]. Substantial evidence confirms that antibiotic preconditioning enhances FMT efficacy by minimizing microbial biomass and diversity in the gut, thereby creating a reduced niche for subsequent engraftment [74,75,76]. A recent real-world cohort study of 1170 CDI patients receiving FMT demonstrated a positive correlation between antibiotic pretreatment duration and clinical success: cure rates were 45% with 1–5 days versus 65% with >30 days [77]. This association was particularly pronounced in antibiotic-refractory CDI cases. The standard antibiotic regimen for patients with CDI is vancomycin (0.5 g, twice daily for 3 days), which enhances the colonization efficiency of donor microbiota by eliminating residual pathogens and reducing microbial load [78].

Although FMT is established as an effective therapy for recurrent *Clostridioides difficile* infection (rCDI), approximately 30% of patients fail to respond to initial FMT in clinical practice [77]. Intestinal biofilms are three-dimensional structures formed by microorganisms (bacteria, fungi, viruses) and their secreted extracellular polymeric substances (EPS, e.g., polysaccharides, proteins, DNA), adhering to the intestinal mucosa or luminal content [79]. These structurally complex and functionally diverse biofilms impair FMT efficacy through multiple mechanisms including antibiotic resistance shielding, engraftment interference, and preconditioning recalcitrance [80,81]. The combination of antibiotics and bowel cleansing enhances overall FMT efficacy [78]. Phage therapy may also target specific strains to disrupt biofilms and potentiate FMT outcomes [82]. Current FMT preconditioning remains dependent on conventional antibiotics such as vancomycin or antibiotic cocktail regimens. However, biofilm eradication necessitates prolonged high-dose therapies that rarely achieve satisfactory resolution and may elevate safety risks [79].

#### 3.1.5. Administration Routes of FMT

Administration routes are categorized into upper and lower gastrointestinal approaches [83]. Upper gastrointestinal delivery modalities include oral capsule administration and nasogastric tube placement, while lower gastrointestinal approaches encompass endoscopic procedures and enema administration. Colonoscopy-guided FMT enables direct delivery of microbial suspensions to the colonic mucosa, ensuring high-dose microbiota engraftment and demonstrating superior efficacy for ulcerative colitis or severe (CDI [84]. However, this invasive approach carries a risk of perforation (albeit rare) and requires bowel preparation, which reduces patient acceptance. Oral capsules offer non-invasive, outpatient-administrable convenience, yet gastric acidity and digestive enzymes may compromise microbial viability, potentially attenuating therapeutic effects [85]. Nasogastric/nasojejunal tubes bypass gastric degradation but pose aspiration risks, particularly in sedated or neurologically impaired patients. Enemas provide low-cost, technically simple administration but are restricted to the distal colon, failing to reach proximal regions where microbiota–gut interactions are critical for conditions like irritable bowel syndrome [86]. Gastroscope-mediated gastroduodenal delivery shares limitations similar to enteric tubes, including procedural discomfort and limited proximal engraftment. Collectively, no single route demonstrates absolute superiority; optimal FMT administration necessitates weighing efficacy, safety, and disease-specific pathophysiological mechanisms.

The therapeutic applications of FMT demonstrate significant potential beyond traditional gastrointestinal interventions. Contemporary research has revealed promising therapeutic implications for neurological disorders, notably Parkinson’s disease. The GUT-PARFECT clinical trial demonstrated significant amelioration of motor symptoms in early-stage Parkinson’s disease patients, as quantified by MDS-UPDRS metrics, concurrent with improvement in associated gastrointestinal symptomatology [16]. Moreover, preclinical studies in AD mouse models have demonstrated FMT’s capacity to enhance cognitive function, attenuate β-amyloid deposition, and suppress tau hyperphosphorylation, collectively contributing to reduced neuroinflammation. These findings suggest the potential therapeutic utility of FMT in AD intervention strategies [14]. FMT represents a therapeutic paradigm that bridges historical medical practices with contemporary scientific advancement. Its emerging applications in AD and other neurological disorders exemplify the convergence of microbiome science and clinical medicine, representing a promising frontier in therapeutic innovation.

### 3.2. Role of FMT in Inducing and Alleviating AD in Mice

Animal studies have preliminarily shown that GM is closely linked to the onset and progression of AD. FMT from AD donors and health donors can deteriorate and improve symptoms and pathological manifestations in animal models, respectively. However, research is still confined to the animal experimental stage, and further investigation and validation are needed before clinical application.

#### 3.2.1. FMT from AD Donors Promotes Disease Progression

**Figure 4:**
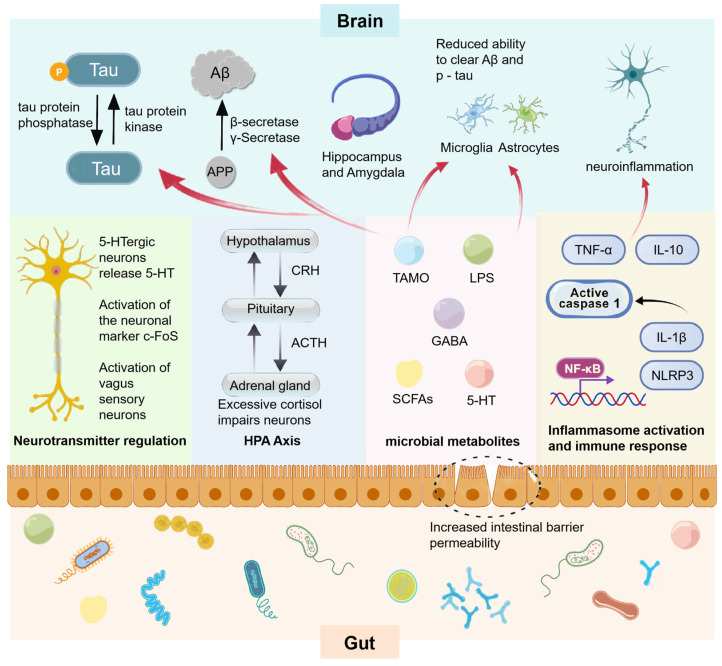
Pathological processes and associations in animal experiments with fecal microbiota transplants associated with AD. Intestinal flora affects related enzymes and immune cells through multiple pathways including neural pathways, endocrine pathways, metabolite pathways, and immune pathways, and promotes the following AD pathological changes in brain structures dominated by the hippocampus and amygdala: (1) Tau protein is phosphorylated under the action of tau protein kinase, resulting in an increase in phosphorylated tau protein (p-tau); (2) The process of amyloid precursor protein (APP) generating amyloid-β protein (Aβ) through the action of β-secretase and γ-secretase is enhanced; (3) The ability of microglia and astrocytes to clear Aβ and p-tau is reduced; (4) The aforementioned processes further induce neuroinflammation. NF-κB activates the transcription of genes such as NLRP3. The NLRP3 inflammasome then activates caspase 1, which processes pro-IL-1β into active IL-1β. Cytokines like TNF-and IL-10 also participate. This drives inflammasome activation and immune response, contributing to neuroinflammation in AD.

Table 1 [87,88,89,90,91,92,93,94,95,96,97] summarizes some FMT animal experiments using AD donors. Transplanting microbiota from AD mice or patients into recipient animals has accelerated AD progression, primarily manifested by Aβ accumulation, hyperphosphorylation of tau protein, enhanced neuroinflammation, abnormalities in glial cells, and impairment of the intestinal wall barrier and the BBB. These pathological changes are interconnected, influencing each other through the MGBA, and collectively exacerbating cognitive impairment (Figure 4).

##### Increment of Brain Aβ Levels

The Aβ hypothesis is a predominant theory for AD etiology. Both Aβ40 and Aβ42 are metabolites of APP, with Aβ42 exhibiting stronger neurotoxicity and a greater propensity to form plaques [98]. The hypothesis posits that increased Aβ production and/or decreased clearance leads to excessive accumulation, particularly of Aβ42, gradually forming oligomers and amyloid plaques [99]. This triggers pathological cascades, including tau hyperphosphorylation, NFTs, synaptic dysfunction, neuronal damage, and ultimately cognitive impairment [100]

Studies have shown that after receiving FMT from *APP/PS1* transgenic mice, young recipient mice exhibited increased brain levels of Aβ38, Aβ40, and Aβ42, along with GM alterations [87]. It was also found that in the fecal microbiota of *APP/PS1* mice, a notable reduction was observed in the phyla *Firmicutes*, *Bacteroidetes*, *Proteobacteria*, and *Actinobacteria*, while there was an increase in *Bacteroides* and *Verrucomicrobia*. At the genus level, *Allobaculum* and *Akkermansia* were found to decrease, whereas *Rikenellaceae* and the *unclassified genus S24-7* were observed to increase. Furthermore, the relative abundance of *Akkermansia* exhibited a negative correlation with brain Aβ42 levels, whereas several bacterial genera showed a positive correlation with brain Aβ42 levels. In a study by Wang et al. [91], 3-month-old *APP*SWE/*PS1*ΔE9 mice that received fecal matter from 16-month-old *APP*SWE/*PS1*ΔE9 mice also demonstrated a significant increase in Aβ plaques, accompanied by inhibited activation of astrocytes surrounding the plaques. These findings suggest that the colonization of GM from AD mice in recipient mice can alter the GM structure and abundance, thereby promoting the progression of AD through increased Aβ pathology in the brain.

##### Increment of Harmful Microbial Metabolites

Trimethylamine N-oxide (TMAO), a metabolite derived from choline, betaine, and carnitine by the GM, has been implicated in promoting neuronal aging, enhancing mitochondrial damage, and increasing superoxide production. It is also associated with increased synaptic damage and a reduction in the expression levels of proteins related to synaptic plasticity by inhibiting the mTOR signaling pathway [101]. Additionally, TMAO has been linked to p-tau and the p-tau/Aβ42 ratio, indicating a potential close relationship with amyloid deposition and axonal injury in neurons [102]. Elevated TMAO levels disrupt blood–brain barrier tight junctions, promote tau hyperphosphorylation (p-Tau Ser396), and activate the NLRP3 inflammasome, exhibiting a positive correlation with mild cognitive impairment (MCI) to AD conversion risk [48].

The transplantation of fecal microbiota from *APP/PS1* mice and AD patients into 6-month-old male C57BL/6J mice led to elevated plasma levels of TMAO and significant endoplasmic reticulum (ER) stress in the brain cortex [94]. The GM of the recipient mice became dysbiotic, with a decrease in *Bacteroidetes* and *S24-7*, and an increase in *Firmicutes*, *Spirochaetaceae*, and *Desulfovibrionaceae*. Elevated phosphorylation levels of cortical proteins and eIF2α indicated excessive ER stress. Another study also found increased levels of histidine and aminohexanoic acid in the recipient mice post-transplantation [93]. These experiments suggest that FMT from the GM of AD patients can increase harmful intestinal microbial metabolites in recipient mice, thereby affecting the gut–brain axis and promoting AD progression.

##### Impact on Neurotransmitter Endocrinology

5-HT signaling, associated with Aβ deposition and APP expression, mediates AD pathology through neuroendocrine pathways [103,104]. Muscarinic acetylcholine receptors (mAChRs), crucial in neurotransmitter regulation, play a significant role in neuronal excitability, synaptic transmission, and neurotransmitter release. A study transplanted feces from Tg2576 mice into AiDM-ICR mice, observing a decrease in 5-HT levels in the mid-colon of the recipients, along with reduced levels of mAChR M2, M3, and Gα proteins [96]. Similarly, a decrease in GABA levels in the intestines of germ-free C57BL/6N mice following FMT from an 82-year-old male AD patient was observed [88]. GABA deficiency can lead to hyperactive prefrontal and hippocampal neurons, compromising cognitive functions such as attention and memory [105]. As neurotransmitters influenced by GM, 5-HT and GABA regulate neural function and affect the CNS. FMT from AD donors can alter the gut ecology of the host, reducing neurotransmitter levels and weakening their regulatory effects on neural function within the MGBA through neuroendocrine pathways, ultimately leading to cognitive impairment.

##### Activation of Inflammatory Vesicles

The NLRP3 inflammasome, a key component of the innate immune system, plays a critical role in resisting pathogen infection and danger signal stimulation. Its activation is closely linked to AD. Transplanting the GM from AD patients into *APP/PS1* double-transgenic mice resulted in increased NLRP3 expression in the intestines and elevated levels of inflammatory factors in peripheral blood. Mice receiving the transplant exhibited more severe cognitive impairments compared to controls that did not receive FMT from AD patients [89].

The NLRP3 inflammasome can be activated by various pathogens and stimuli, such as TLR4 or TNF signaling. This activation initiates the transcription of inflammasome components like NLRP3 and IL-1β via the NF-kB signaling pathway. The active aggregates of these components mediate the activation of caspase-1 and the maturation and release of downstream inflammatory factors, as well as apoptosis [106]. Kim et al. reported increased levels of TNF-α and IL-1β in both the colon and plasma of recipient mice that received FMT from 5xFAD mice [90]. The elevated levels of IL-1β, IL-10, and NLRP3 were also observed in mice after receiving FMT from AD patients [93]. Excessive inflammatory cytokines can lead to a persistent inflammatory response, excessive immunity, and neuronal apoptosis, which can also reach the CNS through blood circulation, deepening neuroinflammation and promoting cognitive decline and AD progression.

##### Abnormal Activation of Microglia

Microglia, which originate from the erythromyeloid progenitors in the yolk sac, are specialized macrophages that perform a variety of critical functions within the CNS. They regulate programmed cell death in neurons, eliminate superfluous synapses during neural development, and foster the formation of new synaptic connections. These cells are essential for preserving tissue homeostasis, coordinating responses to injury, and defending against infections [107]. Microglia engage with neurons and astrocytes to offer nutritional support, react to cytokines and metabolic cues from the neural milieu, and facilitate the optimization of functional neuronal networks [108]. When stimulated, microglia can transition from a quiescent state to either an M1 pro-inflammatory or an M2 anti-inflammatory phenotype, enabling them to engulf and clear away damaged cells, as well as surplus neurons and synapses [109].

In the research conducted by Jin et al. [97], WT mice that received microbiota transplants from 12-month-old *APP/PS1* mice showed elevated levels of Iba1 and iNOS in the cortex and hippocampus of FMT-AD mice. These markers are typically indicative of microglial activation, suggesting an augmentation in the activation state of the recipient microglia. Kim et al. [90] reported that oral administration of fecal matter from 5xFAD mice to C57BL/6 mice resulted in heightened microglial activation. Transplantation of GM from AD donors modifies the recipient’s existing microbial ecosystem, leading to aberrant microglial activation and the release of numerous inflammatory mediators and neurotoxic agents [110]. This process mediates chronic neuroinflammation in the brain, which can damage neurons and synapses, ultimately resulting in cognitive deficits and accelerating the progression of AD [111].

#### 3.2.2. Healthy Donor FMT Relieves Disease Progression

It has been observed that FMT from healthy mice or human volunteers can exert therapeutic effects on AD (Table 2 [14,15,89,112,113,114,115,116]). The levels of Aβ and hyperphosphorylated Tau protein in the recipients’ brains are observed to decline. Additionally, neuroinflammation is mitigated, the secretion of inflammatory cytokines decreases, microglial cells preserve their homeostatic state, and both the gastrointestinal and BBB function properly. As illustrated in Figure 4, the proliferation of beneficial gut bacteria and their metabolites alleviates the progression of AD by modulating various pathways.

##### Reduction Brain Aβ Level

The prevailing approach to AD treatment relies on the Aβ hypothesis [100]. A multitude of drugs and vaccines aimed at Aβ have been developed by researchers. However, the majority have failed to demonstrate the anticipated efficacy during Phase II or III clinical trials [6,117]. In light of the challenges faced by traditional drug development, FMT may present a novel therapeutic avenue for eliminating Aβ and preventing its aggregation. Elangovan et al. [114] performed a 7-day oral gavage FMT using feces from healthy B6SJL WT donor mice on 16 elderly 5xFAD recipient mice. The findings indicated that the FMT mice experienced a decrease in cortical amyloid protein load and quantifiable plaque counts, along with enhancements in cognitive function, new object recognition ability, and spatial memory. Additionally, the reduction in Aβ plaque load was inversely correlated with the trend of cognitive improvement. FMT from healthy donors may have impacted the mechanisms of Aβ clearance or renewal in the amygdala, hippocampus, and other brain structures. Animal studies suggest that FMT from healthy mice can effectively lower brain Aβ levels and successfully ameliorate cognitive deficits, underscoring the considerable potential of FMT in the treatment of AD.

##### Reduces Brain Tau Protein Phosphorylation Level

The hyperphosphorylation of tau protein, which contributes to the formation of NFTs, is a primary pathological hallmark of AD. Research suggests that the chronic inflammatory response in glial cells and neurons is a significant contributor to the hyperphosphorylation of tau protein, and this hyperphosphorylation subsequently amplifies the inflammatory response, further exacerbating AD pathology [118]. The serine/threonine protein phosphatase 2A (PP2A) is the most active phosphatase known for dephosphorylating hyperphosphorylated tau protein back to its normal state [119]. Following dephosphorylation, tau protein can resume its normal function. Experiments have employed carnosic acid (CA) to modulate PP2A and suppress the hyperphosphorylation of tau protein in the brains of *APP/PS1* mice [120]. Recent research has introduced a dephosphorylation-targeting chimera (DEPTACs) strategy, designed to specifically recruit phosphatases to tau to mitigate its hyperphosphorylation as a therapeutic approach for AD. Despite the initiation of numerous clinical trials targeting tau protein, several challenges persist, and many potential drug trials have been halted due to toxicity and/or lack of efficacy [121]. FMT experiments revealed that tau phosphorylation at the threonine 231 site was notably reduced in mice treated with FMT, and cognitive function was enhanced, indicating that FMT can inhibit tau hyperphosphorylation in Tg mice [14]. Additionally, some studies propose that phosphorylated tau at serine 396 and 404 (p-tau396, 404) may act as a therapeutic target for AD, with p-tau396, 404 being more likely than Aβ plaques to induce neuronal damage [122,123,124]. These experiments confirm that FMT from healthy donors can ameliorate cognitive impairment by reducing the abnormal phosphorylation of tau protein at various brain sites.

##### Regulation of Abnormal GM and Its Metabolites

The GM and its metabolites, including TMAO, GABA, SCFA, and endotoxins, play a significant role in the progression of AD through various mechanisms. Dysbiosis of the gut microbiome results in alterations of metabolites that can exacerbate cognitive deficits. Introducing a healthy gut microbiome to restore normal levels of microorganisms and metabolites may alleviate symptoms of AD.

Transferring the GM from WT mice to AD mice reversed the increases of *Firmicutes* and *Prevotella* and the decreases of *Bacteroides* and *Akkermansia* [115]. Furthermore, the abundance of *Vibrio*, *Odoribacter*, and AF12 increased. Following FMT, the AD mice exhibited significant improvements in short-term memory and cognitive abilities, along with a reduction in inflammatory factors in the plasma and a decrease in Aβ plaque load in the hippocampus and cortex. Sun et al. [14] administered a fresh fecal solution from WT mice to *APP*swe/*PS1*dE9 transgenic mice, resulting in a reversal of the AD-related changes in GM and SCFAs levels in the recipient mice, with an increase in SCFAs and a decrease in Aβ40 and Aβ42. Meanwhile, cognitive function also improved. FMT from healthy donors regulated the abnormal composition and abundance of GM in AD mice, reversed the AD-related changes in the gut environment, reduced Aβ plaques, and improved cognitive dysfunction, demonstrating promising therapeutic effects for AD.

##### Reducing the Inflammatory Response

Glycolysis and lipid metabolism are pivotal in the activation of the NLRP3 inflammasome [125]. Curbing aberrant glucose metabolism can attenuate the activation of the NLRP3 inflammasome [126]. Furthermore, certain immune metabolites, including kynurenine and β-hydroxybutyrate (BHB), have been shown to inhibit the activation of the NLRP3 inflammasome [127,128,129]. Studies have suggested that suppressing the activation and expression of the NLRP3 inflammasome can diminish the polarization of microglia towards the M1 phenotype, reduce Aβ deposition and NFT formation, and alleviate neuroinflammation, positioning it as a crucial target for the prevention and treatment of early cognitive dysfunction [130].

A healthy GM can mitigate inflammatory responses by modulating digestive metabolic processes. When healthy human GM was transplanted into *APP/PS1* mice that had previously been colonized with GM from AD patients, a downregulation of NLRP3 expression in the gut was observed, indicating enhancements in cognitive abilities, and a reduction in neuroinflammatory factors [89]. In addition, *APP/PS1* transgenic mice that received FMT from healthy C57BL/6 J mice experienced regulation of their GM composition and abundance, which led to decreased levels of inflammatory factors such as IL-1β and IL-6, as well as reduced Aβ, resulting in improved cognitive function [116]. These animal experiments illustrate that the introduction of GM from healthy donors into mice can suppress the activation of the NLRP3 inflammasome, leading to decreased neuroinflammation and neuronal damage. This, in turn, enhances cognitive function and yields therapeutic benefits against AD.

##### Restoration of Microglia Homeostasis

Microglia play a pivotal role in the pathological progression of AD. Typically, they are responsible for eliminating excess neurons and preserving the equilibrium of the CNS. Nevertheless, they may transition into an aberrant activation state upon stimulation, secreting neurotoxic cytokines that inflict neuronal harm and result in cognitive deficits. Research focusing on microglia has put forth two strategies to ameliorate cognitive dysfunction. One involves restoring microglial equilibrium [131,132], while the other aims at selectively eradicating microglia [133,134]. A multitude of animal model studies suggest that modulating the function of dysfunctional microglia to reestablish normalcy could be a potent approach for eliminating Aβ plaques and preventing AD pathology [135,136]. Healthy human GM was transplanted into *APP/PS1* mice that had previously been given GM from AD patients. The results showed that the activation of central hippocampal microglia was suppressed, and the cognitive abilities of the mice were enhanced [89]. Additionally, the reactivity of microglia in recipient mice was diminished, and a reduction in Aβ plaques and NFTs was observed, accompanied by improvements in cognitive impairment [15]. These animal experiments corroborate that following the recipient’s receipt of FMT from healthy donors, the abnormal activation of microglia can be mitigated, progressively reinstating healthy homeostasis and neuroprotective functions, thereby diminishing neuronal damage and exerting therapeutic effects against AD.

### 3.3. The Role of FMT in Clinical Remission of AD

The application of FMT in the treatment of AD has yielded encouraging outcomes in animal trials, prompting researchers to delve into its potential through clinical studies. To date, there have been several documented cases of FMT being utilized for AD treatment.

Hazan presented the first case study involving an 82-year-old male patient with AD, who also suffered from pneumonia and recurrent CDI in 2020 [17]. This patient underwent FMT from his 85-year-old wife. Remarkably, two months following the procedure, the patient exhibited enhanced mental acuity and emotional well-being, as evidenced by a rise in his MMSE score from 20 to 26. By the fourth month post-FMT, the patient’s memory had continued to improve, and his symptoms had not worsened. By the six-month mark post-procedure, the patient demonstrated substantial emotional progress, improved social interaction, and increased expressiveness, with his MMSE score further improving to 29.

A patient diagnosed with diabetes mellitus, hypertension, chronic kidney disease, and CDI underwent FMT from a healthy 29-year-old male donor. One month subsequent to the initial FMT, her cognitive abilities exhibited a modest enhancement, with her MMSE score increasing from 15 to 18 and MoCA score from 11 to 12. Following one week after the second FMT, MMSE and MoCA scores further improved to 20 and 17, respectively. The patient also reported substantial enhancements in mood and daily functioning, demonstrating a greater range of emotional expression. The report suggested that post-FMT, the patient’s GM displayed elevated levels of certain bacterial genera, such as *Lactobacillus*, *Bacteroides*, *Tannerella*, and *Actinobacteria*, which rectified the microbial imbalance in the recipient and modified the abundance of microbes impacting the MGBA [137].

In a clinical case-control study conducted [138], ten dementia patients with severe CDI who underwent FMT were compared with ten dementia patients with severe CDI treated with antibiotics. The FMT group showed significant improvements in clinical symptoms and cognitive function compared to those treated with antibiotics, accompanied by changes in gut microbiome composition, including increased abundance of *Proteobacteria* and *Bacteroides*, as well as enhancements in the metabolism of alanine, aspartate, and glutamate. This study preliminarily suggests the potential of FMT to alleviate and slow down cognitive decline in dementia patients.

Post-acute coronavirus disease 2019 syndrome (PACS) is a multisystem disorder characterized by a constellation of debilitating symptoms persisting beyond severe acute respiratory syndrome coronavirus 2 (SARS-CoV-2) infection or coronavirus disease 2019 (COVID-19) [139]. PACS typically manifests with long-term debilitating symptoms affecting multiple organs and systems, and is frequently associated with gut microbiome dysbiosis [140]. An in vivo study demonstrated that FMT from PACS patients into germ-free mice—without SARS-CoV-2 exposure—induced pulmonary inflammation, exacerbated pathological lung alterations following multidrug-resistant Klebsiella pneumoniae infection, and triggered cognitive impairment [141]. Furthermore, a prospective interventional study involving 60 PACS patients with insomnia revealed that FMT effectively and safely alleviated post-COVID insomnia and anxiety, improved sleep quality, and reduced daytime hypersomnolence [142].

Dale E. Bredesen and Kenneth Sharlin outlined a cohort study of 100 patients with AD, mild cognitive impairment (MCI), and subjective cognitive impairment (SCI) who achieved cognitive improvement through the personalized precision medicine protocol ReCODE. This approach emphasizes identifying and targeting underlying etiological factors—including pathogen infections, intestinal permeability, insulin resistance, nutritional deficiencies, hormonal deficits, and toxin exposure—with tailored interventions such as dietary modification, gut barrier repair, insulin sensitivity enhancement, hormone replacement, and GM modulation. The study validated the efficacy of multifactorial targeted therapy [143]. These findings underscore the necessity for individualized treatment due to the heterogeneous combination of pathological drivers in each patient’s cognitive decline, thus providing a framework for integrating FMT with other therapeutic modalities to modify AD progression.

In summary, existing clinical studies have shown that FMT can effectively regulate the gut microbiome of patients with AD, and improvements in patients’ clinical symptoms and cognitive functions have been observed in the studies. However, the current scope of clinical trials is limited, mainly focusing on case reports and case-control studies with small sample sizes. To further confirm the clinical efficacy of FMT, more large-scale randomized controlled trials are needed.

## 4. Current Limitations and Perspectives of FMT in the Treatment of AD

Despite promising results in AD treatment, FMT faces several notable limitations and challenges that warrant careful consideration. The primary concern relates to adverse events (AE), ranging from mild gastrointestinal symptoms to severe complications. Common mild adverse events include diarrhea, bloating, and abdominal pain [144]. A case report documented an AD patient experiencing severe gastrointestinal distress following initial FMT, though symptoms improved after subsequent treatment [137]. Of particular concern are immunocompromised individuals and those with inflammatory bowel disease, who have been identified as high-risk populations for FMT-related complications [145]. This was tragically highlighted when the FDA reported fatality in an immunocompromised patient due to multi-drug resistant bacterial infection post-FMT [146]. Research indicates that FMT-related adverse events predominantly occur in patients with compromised mucosal barriers, with the upper gastrointestinal delivery route associated with higher adverse event incidence compared to lower gastrointestinal administration [147].

Long-term safety concerns persist regarding microbiome modification, including potential risks of obesity, metabolic syndrome, autoimmune diseases, and colon cancer [148]. Post-FMT weight gain has been documented in both preclinical and clinical studies [149,150]. While rare, the risk of pathogen transmission remains relevant, with reported cases of post-FMT Norovirus gastroenteritis [151]. To address these safety concerns, both the American Gastroenterology Association’s national FMT registry and China’s FMT platform have initiated comprehensive follow-up studies [152,153]. Methodological challenges in current FMT research include the persistence of indigenous microbiota despite broad-spectrum antibiotic pre-treatment, potentially confounding experimental results [63]. Furthermore, standardization issues persist regarding treatment protocols, dosing regimens, and intervention timing and frequency.

Recent research has revealed promising directions for FMT optimization. Studies demonstrate superior cognitive outcomes and reduced cortical amyloid levels with young versus elderly donors [114], suggesting donor age as a crucial variable for treatment efficacy. Raber’s team discovered that germ-free mice receiving FMT from AD model mice exhibited sex-specific differences in cognitive changes, which were further modulated by specific genotypes (e.g., *App*^NL-G-F/E4^). For instance, male *App*^NL-G-F^ recipients showed impaired novel object recognition, whereas females did not. Among male recipients, those receiving *App*^NL-G-F/E4^ microbiota demonstrated a trend toward a higher percentage of entries into the novel arm of the Y-maze compared to WT recipients. In contrast, donor genotype had no significant effect on the percentage of novel arm entries in female recipients [154]. A 2025 review highlighted that in FMT studies targeting cognitive improvement in hepatic encephalopathy or Parkinson’s disease, several trials reported sex-dependent efficacy (e.g., greater enhancement in executive function among male patients) [155]. However, no randomized controlled trials (RCTs) specifically investigating sex-specific responses to FMT in AD patients currently exist. Existing clinical data primarily derive from small exploratory studies that did not control for gender as a confounding variable. Additionally, targeted microbiota transplantation successfully restored GM composition and improved AD-related pathology using eight dominant species [116]. Future research directions should prioritize the identification and isolation of specific beneficial GM species for more targeted interventions, potentially leading to more precise and effective treatments. Investigation of preventive applications at different AD stages could expand our understanding of optimal intervention timing and potentially establish FMT as a preventive measure against severe AD progression. Integrated multi-omics analyses incorporatingGM, metabolome (e.g., SCFAs, TMAO), proteome (e.g., p-tau, NFL), and miRNA profiles (e.g., hsa-miR-4455) would enable the construction of high-specificity predictive models for AD during its prodromal phase or mild cognitive impairment (MCI) stage. A research project found that hsa-miR-4455 is the best biomarker in all omics analyses. The diagnostic index taking a ratio of hsa-miR-4455 to hsa-let-7b-3p predicted aMCI patients against healthy subjects with 97% overall accuracy [156]. One-hundred-and-sixty-two adults with asymptomatic cardiovascular disease but without diagnoses of cognitive impairment or dementia were evaluated. Phosphorylated tau217 accurately differentiated cognitive impairment in patients with cardiovascular disease [157]. Under the criteria based on relevant biomarkers, FMT can be implemented during the prodromal stage of AD or in patients with mild cognitive impairment (MCI) to better mitigate the progression of AD.

Additionally, optimization of donor selection criteria, particularly regarding donor age, warrants thorough investigation given the demonstrated superiority of young donors in preclinical studies. The development of standardized treatment protocols and delivery methods is crucial for improving reproducibility and clinical outcomes. Furthermore, establishment of long-term safety and efficacy profiles through rigorous clinical trials remains paramount.

## 5. Conclusions

Emerging evidence substantiates the pivotal role of GM dysbiosis in AD pathogenesis through multi-dimensional interactions along the MGBA. Our synthesis demonstrates that FMT exerts therapeutic effects by modulating Aβ metabolism and tau phosphorylation, restoring neuroendocrine equilibrium, attenuating neuroinflammation, enhancing intestinal and blood–brain barrier integrity. While preclinical studies demonstrate FMT’s remarkable potential in alleviating AD pathology, clinical translation requires methodologically rigorous randomized controlled trials to establish efficacy and safety profiles. Critical challenges include donor–recipient compatibility optimization, administration protocol standardization, and long-term outcome monitoring. Future research should focus on mechanistic exploration, technological innovation, personalized strategies, and preventive applications. This evolving therapeutic paradigm bridges ancient medical wisdom with modern systems biology, offering a transformative approach to combat neurodegenerative diseases through ecological restoration of the human microbiome.

## Figures and Tables

**Table 1:** FMT animal experiments with AD donors.

	Donor	Recipient	Transplantation Technique	Results	Ref.
1	12-month-old CONVR*APPPS1* mice	4-month-old GF-*APPPS1* mice	Oral gavage of fecal contents on day 1 and day 4	↑ β38, β40, β42 ↓ NPE and IDE	Harach et al. [87]
2	82-year-old male AD patients	4-week-old germ-free C57BL/6N mice	Oral inoculation	↓ Cognitive function ↓ OLT and ORT ↓ γ-Aminobutyrate, taurine and valine	Fujii et al. [88]
3	AD patients	*APP/PS1* double transgenic mice	Oral gavage	↑ NLRP3 and neuroinflammatory ↑ Activation of microglia	Shen et al. [89]
4	5xFAD mice	C57BL/6 mice	Oral gavage (200 µL for 5 consecutive days)	↑ p21, TNF-α, IL-1β, Microglia activation, ↑ Pro-inflammatory cytokines ↓ Adult hippocampal neurogenesis and BDNF	Kim et al. [90]
5	16-month-old *APP*SWE/*PS1*∆E9 mice	3-month-old *APP*SWE/*PS1*∆E9 mice	Antibiotic cocktails for 2 weeks and FMT for 7 days by oral gavage	↑ Aβ plaques ↓ Astrocyte activation around Aβ plaques	Wang et al. [91]
6	1-year-old WT mice	4-month-old 5xFAD mice	150 μL fecal preparation via oral gavage one time after antibiotic treatment	↑ Serum LPS binding protein ↑ Plaques in the prefrontal cortex ↓ *Firmicutes*	Valeri et al. [92]
7	AD patients	11-week-old male Sprague-Dawley rats	antibiotic treatment for 7 days, 72 h later FMT for 3 days by oral gavage, then twice per week	↑ I L-1β, IL-10, NLRP3 ↑ Histidine, aminoadipic acid, MIF, ↓ IL-4, dendritogenesis of adult-born neurons	Grabrucker et al. [93]
8	AD patients and *APP/PS1* mice	6-week-old Male, C57BL/6 J mice	Antibiotic cocktail for 3 days then FMT by gavage for 2 weeks	↑ TMAO in the cerebral cortex and serum	Wang et al. [94]
9	Aged 3 × TgAD donor female mice	9–12-week-old male and female C57BL/6 mice	By oral gavage to recipient at 24 h after TBI	↑ *Bacteroidetes*, neuroinflammation ↑ Microglia and Astrocytes activation ↓ *Firmicutes*	Soriano et al. [95]
10	Tg2576 mice	AiDM-ICR mice	By gavage for three days(10 g feces in 0.2 mL of a 1 × PBS solution)	↓ 5-HT, mAChR M2, M3 and Gα proteins Dysregulation of the excitatory function of the ENS	Kim et al. [96]
11	12-month-old *APP/PS1* mice	Newly weaned WT mice	Gavaged with fecal supernatant (200 μL per mouse) three times a week	↑ BACE1, Aβ42, Iba1 and iNOS ↓ Short-term spatial memory ↓ Memory for novel object recognition	Jin et al. [97]

The upward arrow (↑) in the table indicates an increase or improvement in certain microorganisms, metabolites, AD pathological manifestations, cognitive abilities, etc., while the downward arrow (↓) indicates a decrease or impairment in the aforementioned experimental results.

**Table 2:** FMT animal experiments with healthy donors.

	Donor	Recipient	Transplantation Technique	Results	Ref.
1	SAMR1 mice	Pseudo germ-free mice	0.2 mL fecal suspension by gavage for 14 days	↑ α diversity and β diversity ↓ Abnormal microbiota	Zhan et al. [112]
2	Age-matched *APPPS1*-21	ABX-treated *APPPS1*-21 male	0.2 mL fecal slurry by gastric gavage daily starting on P25 until sacrifice	↑ Microglial physiology ↓ Aβ pathology	Dodiya et al. [113]
3	WT mice	*APP*swe/*PS1*dE9 Tg mouse model	0.2 mL of fresh fecal solution by gastric gavage once daily for 4 weeks	↑ SCFAs, synaptic plasticity ↓ Aβ40, Aβ42, p-tau, ↓ COX2, CD11b, neuroinflammation	Sun et al. [14]
4	WT mice	ADLP^APT^ transgenic mouse model	Fresh fecal matter for 4 weeks in mice pre-treated with antibiotics	↓ Aβ plaques, NFT, inflammatory monocytes ↓ Glial reactivity, cognitive impairment	Kim et al. [15]
5	Healthy human	*APP/PS1* mice transplanted with GM from AD patients	Oral gavage	↑ Cognitive function intestinal ↓ NLRP3 and neuroinflammatory factors ↓ Activation of microglia in central hippocampus	Shen et al. [89]
6	Healthy B6SJL WT mice	Old (30–32-week-old) 5xFAD recipient mice	Oral gavage for seven days	↑ Cognitive function, novel object recognition, spatial memory ↓ Inflammatory factors, Aβ plaques	Elangovan et al. [114]
7	WT mice	AD mice	By gavage	↑ *Bacteroidetes*, *Bacteroides*, *Sutterella* ↑ *Oscillospira*, *Odoribacter*, AF12 ↑ Short-term memory level and cognitive ability ↓ *Firmicutes* and *Prevotella*	Hang et al. [115]
8	Healthy C57BL/6 J mice	*APP/PS1* transgenic male mice	0.3 mL of fresh fecal matter for five weeks intragastrically	↑ Intestinal microbiota richness and composition ↓ IL-1β, IL-6, APP, Aβ plaques BACE1	Li et al. [116]

The upward arrow (↑) in the table indicates an increase or improvement in certain microorganisms, metabolites, AD pathological manifestations, cognitive abilities, etc., while the downward arrow (↓) indicates a decrease or impairment in the aforementioned experimental results.

## Data Availability

No new data were created or analyzed in this study. Data sharing is not applicable to this article.

## Abbreviations

AD	Alzheimer’s disease
GM	gut microbiota
MGBA	microbiota–gut–brain axis
FMT	fecal microbiota transplantation
CNS	central nervous system
Aβ	amyloid-β
NFTs	neurofibrillary tangles
HPA	hypothalamus–pituitary–adrenal
BBB	blood–brain barrier
SCFAs	short-chain fatty acids
BDNF	brain-derived neurotrophic factor
GIT	gastrointestinal tract
LPS	lipopolysaccharides
TLRs	toll-like receptors
5-HT	5-hydroxytryptamine
GABA	γ-aminobutyric acid
IL-1	interleukin-1
IL-6	interleukin-6
TNF-α	tumor necrosis factor-alpha
iNKT	invariant natural killer T cells
APP	amyloid precursor protein
CDI	*Clostridioides difficile* infection
TMAO	Trimethylamine N-oxide
p-tau	tau hyperphosphorylation
ER	endoplasmic reticulum
mAChRs	Muscarinic acetylcholine receptors
PP2A	protein phosphatase 2A
CA	carnosic acid
DEPTACs	dephosphorylation-targeting chimera
BHB	β-hydroxybutyrate
